# Patient-associated risk factors for severe anemia in patients with advanced ovarian or breast cancer receiving olaparib monotherapy: A multicenter retrospective study

**DOI:** 10.3389/fonc.2022.898150

**Published:** 2022-10-04

**Authors:** Ryota Tashiro, Hitoshi Kawazoe, Kanako Mamishin, Keisuke Seto, Ryoko Udagawa, Yoshimasa Saito, Hironobu Hashimoto, Tatsunori Shimoi, Kan Yonemori, Masahito Yonemura, Hiroyuki Terakado, Toshikatsu Kawasaki, Tetsuya Furukawa, Tomonori Nakamura

**Affiliations:** ^1^ Department of Pharmacy, National Cancer Center Hospital, Tokyo, Japan; ^2^ Division of Pharmaceutical Care Sciences, Keio University Graduate School of Pharmaceutical Sciences, Tokyo, Japan; ^3^ Division of Pharmaceutical Care Sciences, Center for Social Pharmacy and Pharmaceutical Care Sciences, Keio University Faculty of Pharmacy, Tokyo, Japan; ^4^ Department of Pharmacy, National Cancer Center Hospital East, Chiba, Japan; ^5^ Department of Pharmacy, Center Hospital of the National Center for Global Health and Medicine, Tokyo, Japan; ^6^ Department of Medical Oncology, National Cancer Center Hospital, Tokyo, Japan

**Keywords:** olaparib (Lynparza™), severe anemia, risk factor, red blood cell count (RBC), breast cancer susceptibility (BRCA) gene

## Abstract

**Background:**

Olaparib-induced anemia is a frequently occurring complication in patients with advanced ovarian cancer, fallopian tube cancer, or primary peritoneal cancer and is associated with a marked deterioration in patients’ health-related quality of life. This study aimed to clarify patient-specific risk factors for severe anemia in patients with advanced ovarian or breast cancer receiving olaparib monotherapy in a real-world setting.

**Methods:**

This multicenter, retrospective, observational study enrolled consecutively presenting patients with advanced ovarian or breast cancer who received olaparib monotherapy as maintenance or palliative treatment between April 2018 and December 2020 at three participating medical institutions in Japan. The primary endpoint was patient-associated risk factors underlying the onset of grade ≥3 anemia from olaparib treatment initiation to 90 days after treatment. Receiver operating characteristic curves were constructed and univariable and multivariable logistic regression analyses were performed to evaluate the association between patient-associated risk factors and grade ≥3 anemia.

**Results:**

Of 113 patients evaluated in this study, 32.7% (*n* = 37) had grade ≥3 anemia. Multivariable logistic regression analysis revealed that low baseline red blood cell (RBC) count (<3.3 × 10^6^ cells/μL), low baseline hematocrit level (<35%), low baseline hemoglobin level (<11.6 g/dL), and breast cancer susceptibility (*BRCA1/2*) mutation were significantly associated with the onset of grade ≥3 anemia (adjusted odds ratio [OR], 3.39; 95% confidence interval [CI], 1.28–9.62; *P* = 0.017, adjusted OR, 3.63; 95% CI, 1.28–11.64; *P* = 0.021, adjusted OR, 3.89; 95% CI, 1.39–12.21; *P* = 0.014, and adjusted OR, 4.09; 95% CI, 1.55–11.67; *P* = 0.006, respectively).

**Conclusions:**

Our findings suggest that low baseline RBC count, low baseline hematocrit level, and low baseline hemoglobin level might be the patient-associated risk factors for severe anemia induced by olaparib monotherapy. Additionally, *BRCA1/2* mutation was suggested to be a patient-related risk factor for anemia regardless of severity. Therefore, applying these patient-associated risk factors would help classify and screen patients at risk of severe anemia.

## Introduction

According to a leading statistical report regarding global cancer incidence and prevalence, breast cancer in women was the most commonly diagnosed incident cancer (11.7%) and was ranked as the fourth leading cause of cancer-associated mortality (6.9%) among women in 2020. Ovarian cancer was reported to be the 19^th^ most commonly diagnosed incident cancer (1.6%) and the 13^th^ leading cause of cancer-associated mortality (2.1%) (1). Recently, three pivotal phase III clinical trials have revealed that drugs such as olaparib, polyadenosine, and poly (adenosine diphosphate–ribose) polymerase (PARP) inhibitors show a different mechanism of action as compared to the traditional cytotoxic chemotherapy. These findings have greatly changed the clinical outcomes of patients with advanced ovarian or breast cancer (2–4). Currently, olaparib monotherapy is considered the new treatment standard for patients with advanced ovarian or breast cancer (5, 6).

Nonetheless, in clinical practice, olaparib-induced severe anemia is a frequently occurring complication that is associated with a marked deterioration in patients’ health-related quality of life. A phase III clinical trial was conducted on patients newly diagnosed with ovarian cancer who also had breast cancer susceptibility (*BRCA1/2*) mutation. The trial also included patients with primary peritoneal cancer and fallopian tube cancer. The overall incidence rate of anemia after using olaparib as maintenance therapy was 39%, whereas that of grade ≥3 anemia was 22% (2). Similarly, a phase III clinical trial conducted on patients with platinum-sensitive relapsed ovarian cancer with *BRCA1/2* mutation, primary peritoneal cancer, and fallopian tube cancer reported that the overall incidence of anemia after the use of olaparib as maintenance therapy was 43%, whereas that of grade ≥3 anemia was 19% (3). In a phase II clinical trial involving patients with recurrent platinum-sensitive ovarian cancer, irrespective of the *BRCA1/2* mutation, primary peritoneal cancer, and fallopian tube cancer, the overall incidence of anemia was 17%, whereas that of grade ≥3 anemia was 5% when olaparib was used as maintenance therapy (7). Additionally, in a phase III clinical trial on breast cancer patients, the incidence rates of anemia (any grade) and grade ≥3 anemia were 40% and 16%, respectively (4). Taken together, the incidence of olaparib-induced severe anemia was found to be consistent among patients with ovarian and breast cancer. Moreover, a meta-analysis reported that the relative risk of anemia was significantly higher in patients treated with olaparib than in those who received a placebo (8).

Preclinical research has shown that PARP-2-deficient mice exhibited a significant decrease in hemoglobin levels as compared to the wild-type mice (9). This suggests that the detected decrease in hemoglobin levels may be caused by a decrease in the number of erythrocytes and that this mechanism involves the differentiation of immature erythrocytes due to deoxyribonucleic acid damage and apoptosis as mediated by the *TP53* gene.

Fanconi anemia (FA) is an inherited condition associated with developmental abnormalities, bone marrow failure, and increased risk of cancer, with a prevalence of 1–5 per million births. FA is reported to be caused by a disruption in the Fanconi anemia/breast cancer (FA/*BRCA*) pathway. Moreover, 22 genes involved in the FA/*BRCA* pathway have been identified, including *BRCA1* and *BRCA2* (10–13). This implies that when *BRCA1/2* mutations occur, bone marrow failure, resulting in anemia, is likely to occur.

On the contrary, it has been reported that bone marrow suppression caused by traditional cytotoxic chemotherapy leads to anemia, tumor invasion, and bone marrow destruction, which results in anorexia and malnutrition. These have been reported as direct contributory factors in the development of anemia (14). Moreover, hemoglobin, hematocrit, and body mass index (BMI) have been reported as patient-specific risk factors for chemotherapy-induced severe anemia (15). Importantly, the association between patient-associated risk factors and severe anemia in patients receiving olaparib monotherapy has not yet been established. Considering all the facts, we hypothesized that these patient-associated risk factors could predict severe anemia induced by olaparib monotherapy in clinical practice.

Therefore, this study aimed to clarify the patient-associated risk factors for severe anemia in patients with advanced ovarian or breast cancer treated with olaparib monotherapy in a real-world setting.

## Materials and methods

### Study design and patients

This multicenter, retrospective, observational study was conducted at three participating medical institutions: the National Cancer Center Hospital (Tokyo, Japan), the National Cancer Center Hospital East (Chiba, Japan), and the Center Hospital of the National Center for Global Health and Medicine (Tokyo, Japan). Patient data were extracted from the electronic medical records. Data integration and subsequent analyses were performed at the Keio University Graduate School of Pharmaceutical Sciences (Tokyo, Japan). The methodology adopted in this study followed the STROBE guidelines (16). Some methods used in the present study were previously reported by our co-authors (17–19).

The inclusion criteria for the patients were as follows (1): consecutively presenting and aged ≥20 years, with a diagnosis of advanced ovarian or breast cancer and (2) receipt of olaparib monotherapy (300 mg taken orally twice daily) as maintenance or palliative treatment between April 2018 and December 2020.

The exclusion criteria were as follows (1): incomplete data from the patients’ medical records or lack of baseline laboratory data (2); a lower olaparib dosage at therapy initiation (100–250 mg taken orally twice daily) (3); an olaparib treatment period of <90 days; and (4) restarting the treatment after the discontinuation of olaparib monotherapy due to any reason (except resumption after drug withdrawal).

The study protocol was approved by the Ethics Committees of the National Cancer Center (approval number: 2021-052) and the Center Hospital of the National Center for Global Health and Medicine (approval number: NCGM-G-004274-00). The study was conducted in accordance with the Declaration of Helsinki and the Ethical Guidelines for Medical and Health Research involving Human Subjects by the Ministry of Education, Culture, Sports, Science, and Technology and the Ministry of Health, Labour, and Welfare of Japan. The need for written or oral informed consent was waived by the ethics review committees due to the retrospective nature of the study. Accordingly, we used the official website of the hospital to provide an opt-out option (via a web form) rather than directly acquiring written or verbal informed consent from each patient.

### Data collection

Patients’ data were de-identified and anonymously analyzed. We extracted the necessary baseline clinical and demographic data (defined as the most recent blood count within 4 weeks prior to treatment initiation, as well as other clinical data prior to treatment initiation). The following data were collected: age, sex, type of cancer, BMI, Eastern Cooperative Oncology Group performance status (ECOG PS), medical history of chemotherapy, presence or absence of *BRCA1/2* mutation, and routinely available peripheral blood data, including red blood cell (RBC) count and hemoglobin and hematocrit levels. The follow-up period ended on March 31, 2021.

### Endpoints

The primary endpoint was patient-associated risk factors underlying the onset of grade ≥3 anemia. The secondary endpoint was the onset of grade ≥1 anemia. These endpoints were graded in accordance with the Common Terminology Criteria for Adverse Events (v5.0) (20). Furthermore, based on the common criteria range delineated by the Japan Clinical Oncology Group (21), grades 3 and 1 anemia were defined as hemoglobin levels of <8.0 g/dL (for both sexes) and <11.6 g/dL (for females), respectively. In each patient, anemia was evaluated as the least hemoglobin value measured from treatment initiation to 90 days after the completion of olaparib treatment. If no changes were observed in the anemia grade before and after olaparib administration, it was not considered as the onset of anemia.

### Statistical analyses

Patient characteristics were summarized using descriptive statistics, including frequencies and proportions. Receiver operating characteristic curve analyses and Youden’s index were used to determine the optimal cut-off values for continuous variables to predict the onset of anemia (22). Youden’s index was calculated as the maximum value using the following formula: sensitivity - (1 – specificity). We also calculated the positive and negative predictive values (PPV and NPV, respectively).

Univariable and multivariable logistic regression analyses were performed to evaluate the association between patient-associated risk factors and the onset of anemia. Potential explanatory variables reported by several previous studies, specifically baseline RBC count, baseline hematocrit level, baseline hemoglobin level, age, and baseline BMI values, were included as covariates in the univariable and multivariable models (14, 15). Furthermore, we included *BRCA1/2* mutations since they are part of the genes responsible for FA (10–13). We chose to evaluate only four covariates as a multivariable analysis requires at least 10 events per variable to characterize the outcomes reliably (23, 24).

The results are presented as odds ratios (ORs) and 95% confidence intervals (CIs). All statistical analyses were performed using JMP software version 16.2.0 (SAS Institute Inc., Cary, NC, USA). All *P*-values were two-sided, and a *P*-value of <0.05 was considered statistically significant.

## Results

### Patient characteristics

**Figure 1:**
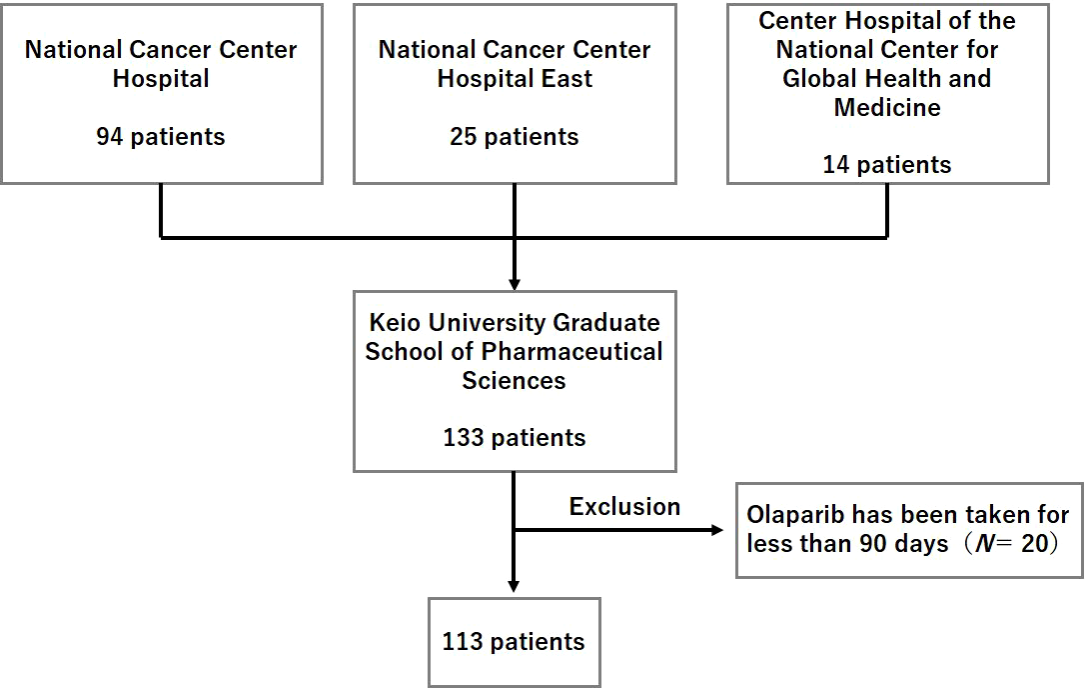
Flowchart illustrating the patient enrollment process.

The patient enrollment flowchart is shown in **Figure 1**. Of the 133 initially identified patients, 20 were excluded from the study based on the aforementioned exclusion criteria. Thus, a total of 113 patients formed the study population.

Patient demographic data are summarized in **Table 1**. All patients were females, and the median age of the enrolled patients was 57 years (interquartile range [IQR], 50–68 years). Olaparib monotherapy was administered to 105 (92.9%) patients with ovarian cancer and eight (7.1%) patients with breast cancer. Moreover, 87 patients (77.0%) were in good condition, with an ECOG PS of 0. The median baseline RBC count was 3.4 × 10^6^ cells/μL (IQR, 3.0–3.7 × 10^6^ cells/μL). The median baseline hematocrit level was 34.2% (IQR, 30.9–36.4%), whereas the median baseline hemoglobin level was 11.4 g/dL (IQR, 10.2–11.9 g/dL). Of all the patients, 44 (38.9%) were positive for *BRCA1/2* mutation, 54 (47.8%) were negative, and 15 (13.3%) had an unknown mutation status.

**Table 1:** Baseline clinical and demographic characteristics of patients.

Characteristics	Patients (*N* = 113)
Sex	
Females, *N* (%)	113 (100)
Age (years), median (IQR)	57 (50–68)
<65 years, *N* (%)	71 (62.8)
≥65 years, *N* (%)	42 (37.2)
Cancer type, *N* (%)	
Ovarian cancer	105 (92.9)
Breast cancer	8 (7.1)
BMI (kg/m^2^), median (IQR)	21.3 (19.5–24.1)
ECOG PS, *N* (%)	
0	87 (77.0)
1–2	26 (23.0)
Previous platinum regimens, *N* (%)	
≤2	89 (73.0)
>2	33 (27.0)
Peripheral blood data	
Baseline RBC count (×10^6^ cells/μL), median (IQR)	3.4 (3.0–3.7)
Baseline hemoglobin level (g/dL), median (IQR)	11.4 (10.2–11.9)
Baseline hematocrit level (%), median (IQR)	34.2 (30.9–36.4)
*BRCA1/2* mutation	
Positive, *N* (%)	44 (38.9)
Negative, *N* (%)	54 (47.8)
Unknown, *N* (%)	15 (13.3)

IQR, interquartile range; BMI, body mass index; ECOG PS, Eastern Cooperative Oncology Group performance status; RBC, red blood cell; *BRCA*, breast cancer susceptibility.

### Endpoints

The incidence of grade ≥3 anemia was 32.7% (*n* = 37), whereas the incidence of grade ≥1 anemia was 61.1% (*n* = 69). The optimal cut-off values for age, baseline RBC count, baseline hematocrit level, and baseline hemoglobin level in predicting the onset of grade ≥3 anemia were initially determined to be 66 years, 3.33 × 10^6^ cells/μL, 33.2%, and 10.8 g/dL, respectively; with corresponding Youden’s index values of 0.126, 0.266, 0.253, and 0.226, respectively. The sensitivity, 1 – specificity, PPV, and NPV for the determined optimal age cut-off value were 78.4%, 65.8%, 29.0%, and 26.0%, respectively. The sensitivity, 1 – specificity, PPV, and NPV for the determined optimal baseline RBC count cut-off value were 62.2%, 35.5%, 23.0%, and 49.0%, respectively. The sensitivity, 1 – specificity, PPV, and NPV for the determined optimal baseline hematocrit level cut-off value were 62.2%, 36.8%, 23.0%, and 48.0%, respectively. Finally, the sensitivity, 1 – specificity, PPV, and NPV for the determined optimal baseline hemoglobin level cut-off value were 56.8%, 34.2%, 21.0%, and 50.0%, respectively. The areas under the curve for age, baseline RBC count, baseline hematocrit level, and baseline hemoglobin level were 0.540, 0.607, 0.634, and 0.622, respectively. Therefore, we decided that an age of 65 years, baseline RBC count, hematocrit level, and hemoglobin level of 3.3 × 10^6^ cells/μL, 35%, and 11.6 g/dL, respectively, were the appropriate cut-off values for further analysis. The optimal cut-off value for BMI was set at 23 kg/m^2^, based on the results of a previous study (15). The median treatment duration was 269 days (IQR, 181–407 days).

The primary endpoints are listed in **Table 2**. Multivariable logistic regression analysis revealed that low baseline RBC count (<3.3 × 10^6^ cells/μL), low baseline hematocrit level (<35%), low baseline hemoglobin level (<11.6 g/dL), and *BRCA1/2* mutation were significantly associated with the onset of grade ≥3 anemia (adjusted OR, 3.39; 95% CI, 1.28–9.62; *P* = 0.017, adjusted OR, 3.63; 95% CI, 1.28–11.64; *P* = 0.021, adjusted OR, 3.89; 95% CI, 1.39–12.21; *P* = 0.014, and adjusted OR, 4.09; 95% CI, 1.55–11.67; *P* = 0.006, respectively). In contrast, high baseline RBC count (≥3.3 × 10^6^ cells/μL), high baseline hematocrit level (≥35%), high baseline hemoglobin level (≥11.6 g/dL), and *BRCA1/2* mutation were significantly associated with the onset of grade ≥1 anemia (adjusted OR, 2.53; 95% CI, 1.02–6.49, *P* = 0.047, adjusted OR, 2.90; 95% CI, 1.11–8.20, *P* = 0.035, adjusted OR, 5.78; 95% CI, 2.10–18.19, *P* = 0.001, and adjusted OR, 2.74; 95% CI, 1.08–7.32, *P* = 0.037, respectively) (**Table 3**).

**Table 2:** Patient-specific risk factors associated with the onset of grade ≥3 anemia.

		Univariable analysis		Multivariable analysis					
Variable		Crude OR (95% CI)	*P*-value	Adjusted OR (95% CI)	*P*-value	Adjusted OR (95% CI)	*P*-value	Adjusted OR (95% CI)	*P*-value
Baseline RBC count	<3.3 × 10^6^ cells/μL	2.84 (1.26−6.40)	0.012	3.39 (1.28–9.62)	0.017				
	≥3.3 × 10^6^ cells/μL	1		1					
Baseline hematocrit level	<35%	2.30 (0.98–5.42)	0.055			3.63 (1.28−11.64)	0.021		
	≥35%	1				1			
Baseline hemoglobin level	<11.6 g/dL	2.24 (0.99–5.33)	0.058					3.89 (1.39−12.21)	0.014
	≥11.6 g/dL	1						1	
Age	<65 years	1.63 (0.70–3.77)	0.256	1.57 (0.57–4.46)	0.385	1.70 (0.62–4.87)	0.309	1.87 (0.68–5.44)	0.236
	≥65 years	1		1		1		1	
BMI	<23 kg/m^2^	1.47 (0.64–3.40)	0.364	2.69 (0.93–8.75)	0.080	2.60 (0.90–8.30)	0.089	2.70 (0.94–8.68)	0.076
	≥23 kg/m^2^	1		1		1		1	
*BRCA1/2* mutation	positive	3.67 (1.51−9.39)	0.005	4.09 (1.55−11.6)	0.006	4.19 (1.58−11.9)	0.005	4.30 (1.62–12.38)	0.005
	negative	1		1		1		1	

Multivariable logistic regression analysis was adjusted for age, BMI, and *BRCA1/2* mutation.

RBC, red blood cell; BMI, body mass index; OR, odds ratio; CI, confidence interval; *BRCA*, breast cancer susceptibility.

**Table 3:** Patient-specific risk factors associated with the onset of grade ≥1 anemia.

		Univariable analysis		Multivariable analysis					
Variable		Crude OR (95% CI)	*P*-value	Adjusted OR (95% CI)	*P*-value	Adjusted OR (95% CI)	*P*-value	Adjusted OR (95% CI)	*P*-value
Baseline RBC count	<3.3 × 10^6^ cells/μL	1		1					
	≥3.3 × 10^6^ cells/μL	1.71 (0.79–3.73)	0.172	2.53 (1.02–6.49)	0.047				
Baseline hematocrit level	<35%	1				1			
	≥35%	1.75 (0.80–3.93)	0.167			2.90 (1.11−8.20)	0.035		
Baseline hemoglobin level	<11.6 g/dL	1						1	
	≥11.6 g/dL	4.17 (1.83−10.13)	0.001					5.78 (2.10−18.19)	0.001
Age	<65 years	1.52 (0.70–3.31)	0.292	1.65 (0.65–4.17)	0.287	1.50 (0.59−3.80)	0.390	1.34 (0.50−3.53)	0.557
	≥65 years	1		1		1		1	
BMI	<23 kg/m^2^	1.24 (0.56–2.75)	0.598	1.63 (0.64–4.14)	0.303	1.67 (0.66−4.30)	0.279	1.59 (0.60−4.27)	0.348
	≥23 kg/m^2^	1		1		1		1	
*BRCA1/2* mutation	positive	3.40 (1.44−8.53)	0.007	2.74 (1.08–7.32)	0.037	2.68 (1.06−7.16)	0.041	2.82 (1.07−7.85)	0.040
	negative	1		1		1		1	

Multivariable logistic regression analysis was adjusted for age, BMI, and *BRCA1/2* mutation.

RBC, red blood cell; BMI, body mass index; OR, odds ratio; CI, confidence interval; *BRCA*, breast cancer susceptibility.

## Discussion

The present study showed that low baseline RBC count, low baseline hematocrit level, and low baseline hemoglobin level might be the patient-associated risk factors for severe anemia induced by olaparib monotherapy. In addition, *BRCA1/2* mutation was suggested to be a patient-related risk factor for anemia regardless of severity. RBC count, hematocrit level, and hemoglobin level are closely related to the hematopoietic function. It is considered that severe anemia is more likely to occur in patients receiving olaparib since this drug is administered during the period when the hematopoietic function is already impaired. To the best of our knowledge, this is the first report on the association between patient-associated risk factors and severe anemia in patients treated with olaparib monotherapy in a real-world setting. Patients with *BRCA1/2* mutations are at risk of FA, which may have affected the frequency and severity of olaparib-induced anemia.

The mechanisms underlying the correlations of low baseline RBC count, hematocrit level, and hemoglobin level with respect to the onset of severe anemia have not yet been fully clarified. When the erythrocyte parameters of PARP-2^-/-^ mice and wild-type mice were compared in a preclinical setting, the RBC count, hematocrit level, and hemoglobin level were significantly lower in the PARP-2^-/-^ mice than in the wild-type mice (9). The results of the present study were in concordance with the findings reported in the aforementioned studies. Razzaghdoust et al. reported that in 305 patients with solid tumors or lymphoma who received traditional cytotoxic chemotherapy, hemoglobin level, hematocrit level, and BMI values were strong predictors of severe anemia (15). Aligning with this study, the present study demonstrated that hematocrit level complemented the prediction of severe anemia. BMI was also associated with the incidence of severe anemia. The patients’ backgrounds in the present study differed from patients enrolled in prior investigations. However, we believe that the mechanisms underlying the association between patient-associated risk factors and severe anemia are common between olaparib and traditional cytotoxic chemotherapy. Although we did not investigate mean corpuscular volume in this study, which is a part of the hematopoietic parameters, it may be a risk factor for olaparib-induced anemia as well as an explanatory factor for other hematopoietic parameters. Additionally, no patients with myelodysplastic syndrome or acute myeloid leukemia were included in the study. The prevalence of FA is 1−5 per million live births (10–13). Therefore, the effect of *BRCA1/2* mutations on anemia is not significant.

To the best of our knowledge, the present study is the first to indicate that low baseline RBC count, low baseline hematocrit level, and low baseline hemoglobin level could be patient-associated risk factors for severe anemia induced by olaparib monotherapy. Moreover, the present study is also the first to identify *BRCA1/2* mutation as a possible patient-related risk factor for anemia regardless of severity. These parameters are easily obtainable and do not require additional cost or time. Applying these predictors would help classify and screen patients at high risk for olaparib-induced severe anemia in clinical practice.

The incidence of anemia in the present study was higher than that in previous studies. Olaparib is reported to be metabolized by CYP3A4/5. It is reported that the incidence of CYP3A5 differs between Japanese and European and United States patients, which may have affected the results of this study (25). Moreover, unlike in Europe and the United States, specific standards for dietary folic acid intake have not been established in Japan, except during pregnancy. Additionally, there may be a dissociation between the characteristics of patients in clinical trials and those in actual clinical practice, which may have also affected our results.

Previous studies have shown that prophylactic erythropoietin reduces the negative consequences of chemotherapy-induced severe anemia and improves the patients’ health-related quality of life (26–28). However, this practice is considered an off-label use in Japan. A previous study reported that erythropoietin production is increased in a compensatory manner in PARP-2^-/-^ mice and contributes to the maintenance of erythropoiesis, thus suggesting that erythropoietin may have an effect on ameliorating the risk of severe anemia (9). Considering all these facts, oncologists and other treating clinicians can provide personalized, evidence-based clinical management for these high-risk patients.

High baseline RBC count (≥3.3 × 10^6^ cells/μL), high baseline hematocrit level (≥35%), and high baseline hemoglobin level (≥11.6 g/dL) were significantly associated with the onset of grade ≥1 anemia. The reason for this finding may be attributed to the criteria for evaluating anemia. In the present study, patients who did not show a change in their anemia grade before and after olaparib administration were not considered to have new onset anemia. This result suggests that progression from grade 0 to grade 1 anemia is more likely to occur than a progression from grade 1 to grade ≥2 or from grade 2 to grade ≥3.

The present study has two strengths. First, this was a multicenter study involving three participating medical institutions in Japan, including the National Cancer Center. Therefore, our data may be generalizable to similar populations in the clinical setting. Second, we focused on olaparib monotherapy for patients with advanced ovarian or breast cancer since olaparib monotherapy has become the new treatment regimen for such patients.

Nevertheless, we acknowledge some limitations in our study. First, this was a retrospective observational study. Thus, we could not exclude the possibility of information bias. However, we conducted a multivariable analysis to reduce the effects of biases inherent in observational studies and potential confounders that may be associated with clinical variations in patient characteristics. However, by definition, we could not control the unmeasured confounders during the multivariable analysis. Second, the sample size of the current study was modest; therefore, we could not include more than four covariates in the multivariable analysis. The baseline RBC count, hematocrit level, and hemoglobin level seemed to be correlated. However, in the preclinical study, RBC, hematocrit, and hemoglobin were decreased in PARP-2-deficient mice compared to wild-type mice (9); therefore, we selected these as explanatory variables. While the mechanism of olaparib-induced anemia is not clear, we believe it is important to analyze all of these entities as explanatory variables. Third, we only enrolled patients with advanced ovarian or breast cancer. In recent years, olaparib has been approved for patients with breast cancer possessing mutated *BRCA1/2* gene, male patients with metastatic castration-resistant prostate cancer, and patients with metastatic pancreatic cancer in Japan (29, 30). Fourth, we neither investigated the presence or absence of therapeutic drugs for anemia nor evaluated blood transfusion as a study covariate. Moreover, risk factors for anemia in combination with other drugs (such as bevacizumab) were not investigated, and the period between previous platinum-based chemotherapy and the initiation of olaparib monotherapy was not considered. Furthermore, the presence of grade ≥3 anemia, thrombocytopenia, or neutropenia at the time of the previous platinum-based chemotherapy was not considered. No differences in neutrophil and platelet counts were found between PARP-2-deficient mice and wild-type mice in the *in vivo* study (9). Therefore, we limited the explanatory factors in this study to erythrocyte parameters. Olaparib monotherapy is used as maintenance therapy and is most likely to be prescribed within 1 month of concluding chemotherapy. Fifth, since this study was a retrospective study based on clinical practice, we neither investigated the erythrocyte volume by examining the relationship of the severity of anemia with the duration of olaparib administration nor evaluated the flow cytometry of peripheral blood, which may have revealed incipient myelodysplastic syndrome. Finally, the effect of the olaparib dose on anemia could not be assessed since the dose intensity was not calculated. Overall, our findings should be confirmed using an adequate sample of patients with the above-mentioned solid malignancies who were treated with olaparib.

In conclusion, our findings suggest that low baseline RBC count, low baseline hematocrit level, and low baseline hemoglobin level might be the patient-associated risk factors for severe anemia induced by olaparib monotherapy. Additionally, *BRCA1/2* mutation was suggested to be a patient-related risk factor for anemia regardless of severity. Moreover, early detection of patients at high risk for developing severe anemia can prompt cautious monitoring and optimize the treatment benefit for patients treated with olaparib monotherapy. The findings of the current study can likely be generalized to other populations, thus highlighting the need for additional research in this field.

## Data availability statement

The raw data supporting the conclusions of this article will be made available by the authors upon request, without undue reservation.

## Ethics statement

The studies involving human participants were reviewed and approved by the Ethics Committees of National Cancer Center (approval number: 2021-052) and the Center Hospital of the National Center for Global Health and Medicine (approval number: NCGM-G-004274-00). Written informed consent for participation was not required for this study in accordance with the national legislation and the institutional requirements.

## Author contributions

RT, HK, YS, HH, MY, and TN: conceptualization and design. RT, KM, and KS: data acquisition and patient management. RT and HK: data analysis and interpretation. RT and HK: writing, reviewing, and revising the manuscript. TN: study supervision. All authors contributed to the article and approved the submitted version.

## Funding

This research received no specific grant from any funding agency in the public, commercial, or not-for-profit sectors.

## Acknowledgments

We are grateful to all patients and medical staff at the National Cancer Center Hospital, the National Cancer Center Hospital East, and the Center Hospital of the National Center for Global Health and Medicine who were involved in this study. We would like to thank Editage (www.editage.com) for English language editing.

## Conflict of interest

HK received research funding from Eli Lilly. KM received payment for presentations and expert testimony from Daiichi Sankyo and payment for presentations from Merck Serono. HT received research funding from AbbVie. TN received research funding from Astellas Pharma, Chugai, Daiichi Sankyo, Kyowa Kirin, Otsuka Pharmaceutical, Sanofi, Sato Pharmaceutical, and Shionogi.

The remaining authors declare that the research was conducted in the absence of any commercial or financial relationships that could be construed as a potential conflict of interest. Moreover, the above funders had no role in the design, conduct, or reporting of this investigation. Thus, the authors have no actual conflicts of interest to declare.

## Publisher’s note

All claims expressed in this article are solely those of the authors and do not necessarily represent those of their affiliated organizations, or those of the publisher, the editors and the reviewers. Any product that may be evaluated in this article, or claim that may be made by its manufacturer, is not guaranteed or endorsed by the publisher.

## Abbreviations

BMI, body mass index; *BRCA*, breast cancer susceptibility; CI, confidence interval; ECOG PS, Eastern Cooperative Oncology Group performance status; FA, Fanconi anemia; IQR, interquartile range; NPV, negative predictive value; OR, odds ratio; PARP, poly (adenosine diphosphate–ribose) polymerase; PPV, positive predictive value; RBC, red blood cell.
